# Improvement of Alcohol-Poisoning Symptoms in Mice by the Oral Administration of Live *Lactobacillus plantarum* SN13T Cells

**DOI:** 10.3390/ijms21051896

**Published:** 2020-03-10

**Authors:** Masafumi Noda, Masafumi Maruyama, Narandalai Danshiitsoodol, Fumiko Higashikawa, Masanori Sugiyama

**Affiliations:** 1 Department of Probiotic Science for Preventive Medicine, Graduate School of Biomedical and Health Sciences, Hiroshima University, Kasumi 1-2-3, Minami-ku, Hiroshima 734-8551, Japan; bel@hiroshima-u.ac.jp (M.N.); naraa@hiroshima-u.ac.jp (N.D.); fumiko@hiroshima-u.ac.jp (F.H.); 2 Chugoku Jozo Co., Ltd., Sakurao 1-12-1, Hatsukaichi, Hiroshima 738-0004, Japan; maruyama@chugoku-jozo.co.jp

**Keywords:** alcohol-poisoning symptoms, *Lactobacillus plantarum*, live lactic acid bacteria

## Abstract

A clinical study carried out previously by our group has demonstrated that yogurt manufactured with a plant-derived lactic acid bacterium, *Lactobacillus plantarum* SN13T, significantly reduces the γ-glutamyl transpeptidase (γ-GTP) level as a liver-function parameter. In the present study, we show that with the oral administration of live SN13T cells, alcohol-poisoning symptoms in mice are improved, and the condition does not become fatal. However, prior to the simultaneous administration with ethanol, when the cells were heat-killed or sonicated, the improvement was not observed, and almost all of the mice died. In addition, the dysbiosis of the intestinal microbiota observed in the mice administered with ethanol was restored by simultaneous administration with live SN13T cells. Furthermore, by analyzing the metabolites detected in contents from the mouse cecum, it was demonstrated that the increase in nonvolatile putrefactive amines observed in the ethanol-administration group was reduced by simultaneous administration with live SN13T cells. Judging from these results, the lactic acid bacterial cells capable of reaching the living bowels prevent ethanol-induced poisoning and restore the intestinal microbiota.

## 1. Introduction

Beer and wine are alcoholic beverages that have been drunk since 3000 and 6000 B.C., respectively [1]. These beverages not only are consumed as “pleasure products” but also have been associated with festive rituals and used for medicinal purposes [2]. However, it is obvious that the abuse of alcohol has a close connection to health problems such as hepatitis, pancreatitis, and alcoholism.

Alcoholic steatohepatitis is a hepatic disease caused by the intake of alcohol for a long time. Almost all of the alcohol absorbed into the human body is metabolized by alcohol dehydrogenase (ADH:EC1.1.1.1), which is present mainly in the liver. The enzyme facilitates the interconversion between alcohols and aldehydes or ketones. Although the biological half-life of acetaldehyde is relatively short, in the case of excessive and chronic alcohol consumption, the carcinogenic metabolite acetaldehyde easily accumulates in the human body and finally causes hepatocellular injury [3,4].

Recent studies have shown that ethanol metabolism leads to the inhibition of fatty acid oxidation, as well as the stimulation of lipogenesis, resulting in hepatic fatty acid overload [5]. Up to 20% of heavy alcohol drinkers will suffer from the liver cirrhosis [6], which is a late stage of chronic severe hepatic inflammation defined as the histological development of “regenerative nodules surrounded by fibrous bands” [7].

Lactic acid bacteria (LAB) are generally considered as “probiotics.” The word “probiotics” is defined as “the live microorganisms conferring a health benefit on the host when administered in adequate amounts” [8], and probiotic LAB strains traditionally have been used to manufacture fermented foods. It has been reported that some LAB cells and fermented foods containing the bacteria have potent health benefits, such as promoting intestinal homeostasis, possessing anti-allergic properties, and preventing and improving obesity [9,10,11,12,13,14,15,16].

The LAB strains are roughly classified into two groups: One is designated as animal-derived LAB and have been widely used to manufacture dairy products, such as yogurt and cheese. Another group is designated as plant-derived LAB, which is isolated from plant sources such as fruits, vegetables, flowers, and medicinal plants. We have previously claimed that, when compared with the animal-derived LAB, the plant-derived ones are superior in tolerance against gastric and bile juice, and immunomodulatory activity [17]. We have already isolated over 1000 LAB strains from many plant sources and stored them in a plant-derived LAB library. We have found that some of the stored LAB strains may be useful for preventive medicine, such as immune modulation, reduction of obesity, and anti-allergy [17,18,19,20]. Interestingly, the yogurt manufactured with *Lactobacillus* (*Lb.*) *plantarum* SN13T stored in the library has been demonstrated through a previous clinical study to significantly reduce the serum γ-glutamyl transpeptidase (γ-GTP) value [21].

The aim of this study is to find another healthcare function of the SN13T strain through animal experimentation using mice fed with ethanol. When live SN13T cells were orally administered to mice fed with a diet containing ethanol, death caused by the intake of ethanol was completely avoided, demonstrating that recovery from alcohol-poisoning symptoms in mice was observed only with oral administration of the live cells. In this study, we also demonstrate fluctuations in the intestinal microbiota and metabolites, which are restored by administration of the live cells.

## 2. Results

### 2.1. Health Benefit of Live SN13T Cells against the Alcohol-Poisoning Symptom in Mice

**Figure 1:**
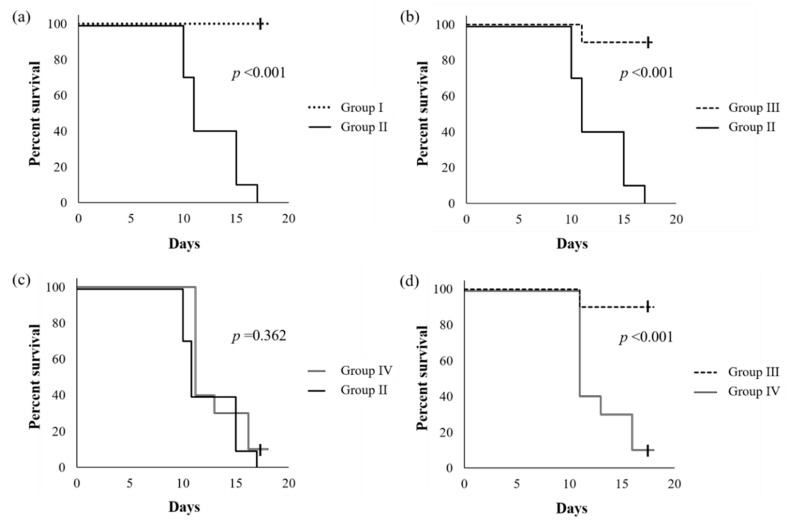
The Kaplan–Meier survival curves of C57BL/6J mice fed an ethanol-containing diet with or without the addition of live SN13T cells. The group fed without ethanol was also compared. The *p*-values were calculated using the log-rank test. Group I, which was fed only an L10015 diet without the administration of ethanol; group II, which was fed an L10016 diet with ethanol; groups III and IV, which were fed with the simultaneous administration of ethanol and the live or heat-killed SN13T cells, respectively, in the L10016 diet. Each graph shows the comparison of survival rate (%) between Groups I and II (**a**), II and III (**b**), II and IV (**c**), and III and IV (**d**), respectively.

Figure 1 shows the survival curves of mice reared using an ethanol-containing diet supplemented with or without SN13T cells. When compared with a group without the intake of ethanol, the cumulative survival rate of the ethanol-intake group without the added live SN13T cells was significantly decreased (*p* < 0.001, Figure 1a), and the mice died within 17 days. However, with simultaneous administration of live SN13T cells, the survival rate of the mice did not decrease when compared with a group receiving no alcohol (Figure 1b), strictly, although only one mouse died. In contrast, the survival rate (*p* = 0.362, Figure 1c) was not improved under supplementation with the heat-killed cells, indicating that there is an obvious difference between the live and heat-killed cell groups (*p* < 0.001, Figure 1d). Thus, the administration of live SN13T cells is essential for improving alcohol-poisoning symptoms.

### 2.2. Fecal Microbiota Analysis Using the Terminal Restriction Fragment Length Polymorphism (T-RFLP) Method

**Figure 2:**
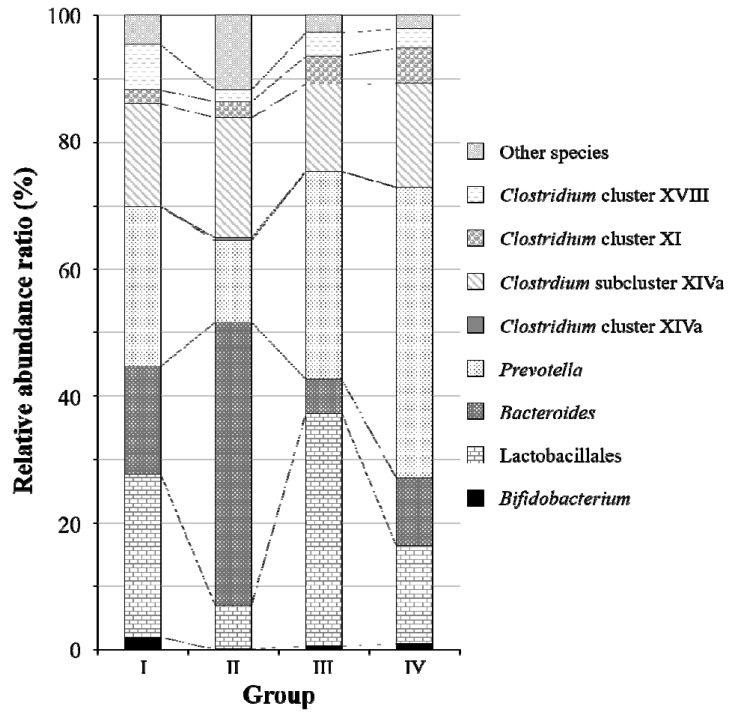
Differences in the fecal microbiota composition in mice after the experimental period as determined via the terminal restriction fragment length polymorphism (T-RFLP) analysis. The experimental content of each group is the same as that in Figure 1.

To evaluate the lethal effect of ethanol and the change in intestinal flora in mice due to the SN13T cells, the intestinal microbiota in feces collected from each group with or without the SN13T cells was analyzed using the T-RFLP method with partial 16S rDNA (Figure 2). When compared with a control group (group I) without the administered ethanol, the relative abundance ratios of the genus *Bacteroides* and the order Lactobacillales were remarkably increased and decreased, respectively, by the administration of ethanol (group II). Specifically, the genus *Bacteroides* was observed to occupy half of the intestinal flora in group II. The changes in *Bacteroides* and Lactobacillales were notably and slightly repressed by the ethanol-containing diet supplemented with live and heat-killed cell groups (groups III and IV), respectively. In addition, the intestinal bacteria, which cannot be classified via the T-RFLP method, were significantly increased in group II.

Based on the T-RFLP analysis, it is suggested that (1) ethanol caused the intestinal microbiota disorder, and (2) supplementation with live SN13T cells improves the microbiota disorder in mice. Although live SN13T cells reach the intestine alive and reduce the lethal function of ethanol, further information is needed to clarify the relationship between lethality and the mechanism of lethality prevention observed only with the live SN13T cells.

### 2.3. Intestinal Microbiota Analysis

**Figure 3:**
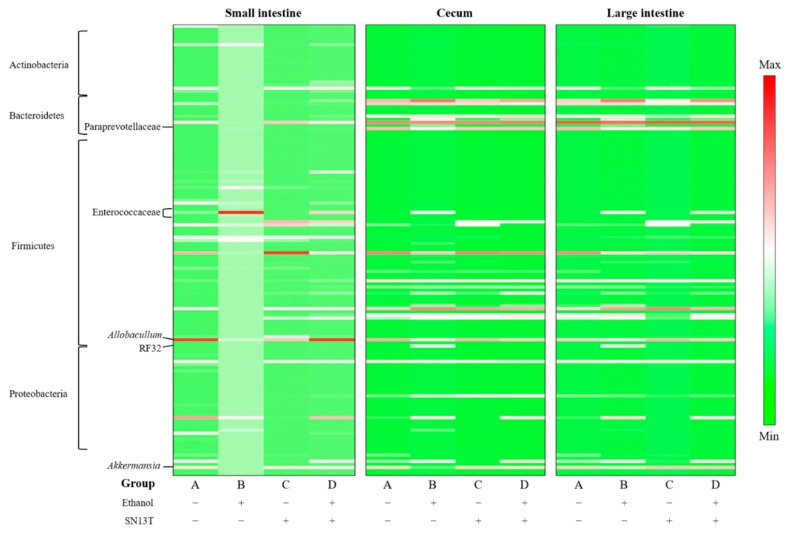
The microbiota in the small intestine, cecum, and large intestine collected from C57BL/6J mice. Prior to the administration of ethanol, the mice were kept for one week using the L10015 diet supplemented with (groups C and D) or without (groups A and B) live SN13T cells. After one week, the L10015 diet was changed to an L10016 diet in groups B and D. The intestinal bacteria were determined using the V3–V4 region in 16S rDNA and indicated by the standardized relative abundance ratio. Denser green and red indicate lower and higher amounts, respectively.

**Figure 4:**
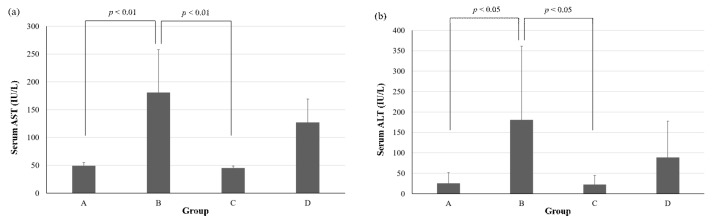
The difference in the serum the aspartate transaminase (AST) (**a**) and alanine aminotransferase (ALT) (**b**) levels of mice with the simultaneous administration of ethanol with or without live SN13T cells. The values are indicated as the mean ± SD. Statistical analysis was performed using the Tukey–Kramer multiple comparisons test. The experimental content of each group is the same as that in Figure 3.

In the present study, we also analyzed the intestinal microbiota of mice fed an ethanol-containing diet supplemented with or without live SN13T cells. As shown in Figure 3, the intestinal microbiota from mice fed the ethanol-containing diet without bacterial cells (group B) was obviously different from that of mice fed a diet supplemented without both ethanol and the bacterial cells (group A). Specifically, with respect to the group B microbiota in the small intestine, Enterococcaceae was extremely increased, and this phenomenon was also observed in the cecum and large intestine. In the cecum, the ratios of *Akkermansia*, *Allobaculum*, and Paraprevotellaceae were remarkably decreased by the administration of ethanol, but these phenomena disappeared with the simultaneous administration with live SN13T cells. On the other hand, although the ratio of an RF32 order was notable increased in group B, interestingly, when live SN13T cells were administered simultaneously with ethanol, the population of RF32 was clearly decreased, as summarized in Table 1. Additionally, the administration of ethanol was found to increase the aspartate transaminase (AST) and alanine aminotransferase (ALT) levels in serum, whereas both levels were reduced by the simultaneous administration of live SN13T cells (Figure 4).

### 2.4. Analysis of Metabolites in the Cecum

**Figure 5:**
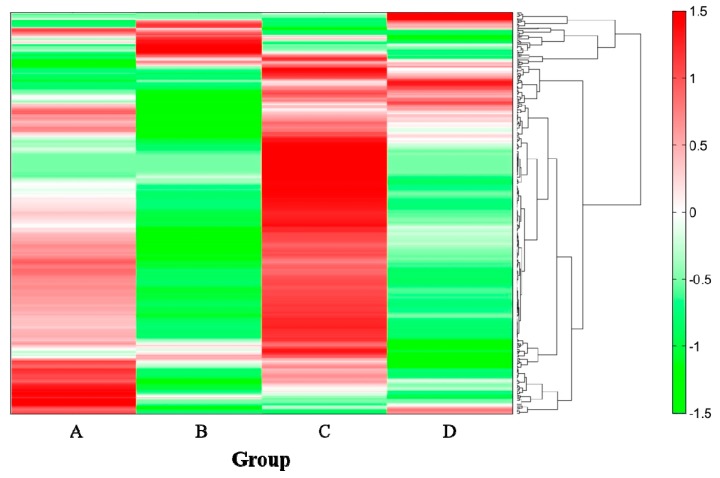
The hierarchical cluster analysis (HCA) of the mouse cecum metabolites. Denser green and red indicate low and high concentrations of metabolites, respectively. The dendrogram depicts the unweighted pair-group method of analysis (UGPMA) based on a standardized relative area of detected compounds. The experimental content of each group is the same as that in Figure 3.

The heatmap obtained from hierarchical cluster analysis (HCA) shows that the metabolite profiles in the cecum were clearly different between ethanol-fed and nonethanol-fed groups (Figure 5). In total, 380 compounds were annotated as metabolites, and 17 of them were found to be increased by the intake of ethanol: Pyruvic acid, propionic acid, 1,3-diaminopropane, stearoyl ethanolamide, 5α-pregnan-3α-ol-20-one, cadaverine, isobutyric acid, butyric acid, *N*-acetylglutamic acid, *o*-hydroxybenzoic acid, tyramine, *N*^1^,*N*^8^-diacetylspermidine, isovaleric acid, valeric acid, isopropanolamine, 2-hydroxy-4-methylvaleric acid, and 2-oxoglutaric acid (Table 2). On the other hand, amino acids were decreased by the administration of ethanol (group B), and the changes did not fully recover. The metabolite analysis shows that some compounds were reduced in group B but not in group D. The detailed HCA result is shown in Supplementary Table S1.

## 3. Discussion

γ-GTP (E.C. 2.3.2.2) is an enzyme that catalyzes the hydrolysis of γ-glutamylpeptide and transfers the γ-glutamyl residue to other peptides and amino acids. The enzyme is induced in a human body that suffers from hepatic and biliary tract diseases. Since the over-induced enzyme flows out from the organs to the blood as a deviation enzyme, the γ-GTP level in serum is used as a biomarker for hepatocellular injury caused by obesity and alcohol abuse.

In our clinical study carried out previously, we have shown that the oral administration of live SN13T cells reduces the serum γ-GTP level [21], suggesting that the LAB cells may improve liver function and protect against alcoholic disorder. Since the C57BL/6J mice have been shown to be useful to investigate the function of alcohol and narcotics, including morphine in previous studies [22,23,24,25], we used the same model of mice to evaluate the toxic effect of alcohol.

As described in a report from the Food and Agriculture Organization of the United Nations (FAO) Nutrition Meeting [26], when mice were continuously fed with 0.8%–20% ethanol, their mortality rate increased in proportion to the ethanol concentration. Acute and high ethanol consumption disrupts the intestinal mucosal barrier and eases the flow of harmful compounds, such as endotoxins, into the body [27]. As a result, hepatic disorder followed by sepsis may induce the invasion of intestinal bacteria into the body [27,28,29,30,31]. These reports support that the death observed in mice fed with ethanol may be due to the dysfunction of the intestinal barrier rather than the alcoholic hepatic disorder. The result obtained in the present mice study reveals that the oral administration of live SN13T cells prevents ethanol-induced poisoning and restores the intestinal microbiota (Figure 1 and Figure 2).

In the microbiota and metabolite analyses, live SN13T cells were administered to the mice prior to ethanol feeding to enhance the preventive effect. Furthermore, to avoid a failure in collecting fresh blood and tissues by sudden mouse death, the mice keeping was terminated when the first death was observed in the ethanol-diet group. This experimental plan allowed us to compare the serum AST and ALT levels in each group. As shown in Figure 4, it was confirmed that live SN13T cells reduce both levels.

Intestinal microbiota analysis in the present study showed that the significant dysbiosis in the mouse small intestine is caused by the intake of ethanol; specifically, the family Enterococcaceae is drastically increased. On the other hand, the dysbiosis of the intestinal microbiota in the cecum and large intestine was not observed, suggesting that the intestinal microbiota has been buffered in the passage of the cecum, because a branched cecum appendix is an apparent safe niche for intestinal bacteria. Thus, the appendix maintains the microbial biofilm composed of indigenous bacteria [32,33]. Considering these results, the microbiota recovery effect observed in the cecum is likely to be important to protect the large intestine from some undesirable bacteria.

Although the significance of the cecum has not been completely elucidated, however, there are significant reports regarding the cecum: That is, surgical removal of the appendix in mice induces the reduction of immunoglobulin A (IgA)-secreting cells in the large intestine and reduces alteration of the fecal microbiota composition [34]. These results indicate that a lymphoid organ in the appendix is necessary to generate and migrate IgA-secreting cells and to regulate the intestinal microbiota. In addition, a comparison study of 533 mammalian species showed that there is a relationship between appendix presence and the concentration of cecal lymphoid tissue [35]. Therefore, the cecum seems to be important for maintaining not only the microbiota balance but also the immune system.

It is still unclear why the ratio of some bacteria, including Enterococcaceae and *Allobaculum* spp., was remarkably changed by the intake of ethanol. It is necessary to determine how these bacteria function or communicate to some microorganisms originally present in the intestine. However, *Akkermansia* spp. and a putative order of RF32 have been shown to be involved in the intestinal inflammatory response. The genus *Akkermansia* consists of two species, *Akkermansia* (*A.*) *glycaniphila* and *A. muciniphila*. The latter species is an anaerobic intestinal commensal bacterium and occupies 1%–4% of the fecal microbiota in humans [36,37]. *A. muciniphila* promotes intestinal barrier function, and the bacterium is rarely present in the intestine of IBD patients [38,39,40]. Furthermore, it has been shown that patients with alcoholic steatohepatitis exhibit a decreased amount of the bacterial species as compared with healthy persons, and the amount of the species in fecal content was also decreased in ethanol-fed mice [41]. On the other hand, there is an obvious relationship among colonic damage, inflammation, and abundance of the RF32 order [42]. Those reports support our hypothesis that the intake of live SN13T cells restores the inflammation caused by ethanol abuse via changing the intestinal microbiota.

Changes in the cecum components, which have a buffering effect against alteration of the intestinal microbiota, may affect the homeostasis. The metabolite analysis done in the present study showed that putrefactive amines, such as tyramine and cadaverine, were increased in the ethanol-fed group. In addition to putrefactive amines, a rise in the volume of isovaleric acid and valeric acid content was also observed. These compounds are generated by the hydrolysis of protein in the putrefaction process of tissues and organs and cause an offensive odor. Further, the observed fall in amino acid volume also indicates the presence of bacteria that hydrolyze proteins and metabolize amino acids. These results reveal that the production of compounds related to putrefaction was promoted during ethanol abuse. Therefore, that putrefaction and the observed mouse death are considered to be due to ethanol abuse. The undesirable effects were repressed by the intake of live SN13T cells. Although it is still not understood what species and reactions are involved in the putrefaction with ethanol, further studies on the indigenous bacteria that belong to putative categories, such as the RF32 order, will be useful to confirm the mechanisms.

In the present study, we demonstrated that alcohol-poisoning symptoms are improved by the oral administration of live SN13T cells but not by the administration of heat-killed cells. Based on these results, it is presumed that live SN13T cells or their collaboration with other intestinal bacteria may generate bioactive compounds. The bioactive compounds may cooperatively inhibit the synthesis of harmful products in the intestinal tract. Some research has shown that a change in the intestinal microbiota is involved in lifestyle and mental diseases [43,44]. We demonstrated in this study that the live SN13T cells are significant for maintaining intestinal microbiota balance and restoring from the symptoms of alcoholism.

## 4. Materials and Methods

### 4.1. Culture Conditions of the LAB Strain

De Man, Rogosa, and Sharpe (MRS) broth (Merck KGaA, Darmstadt, Germany) was used as a medium to cultivate for *Lb. plantarum* SN13T. The bacterium was grown at 37 °C overnight in the MRS broth. After cultivation, the bacterial cells were collected by centrifugation, resuspended, and diluted to the given concentration with sterilized water.

### 4.2. Preparation of Diet for Mice

As a diet with or without added ethanol, L10016 and L10015 (Research Diet, New Brunswick, NJ, USA) were prepared from Pre-Mix L10016A using ethanol or maltodextrin 42, respectively, according to the manufacturer’s protocol, with a slight modification. To evaluate the survival rate of the mice, 6.6% (*v/v*) ethanol at the final concentration or 118.27 g/L maltodextrin 42 was added to Pre-Mix L10016A. If necessary, live SN13T cells (final 1–2 × 10^9^ cfu/mL) were added to the L10016 diet.

To analyze the microbiota or metabolites of the mice, 7.5% (*v/v*) ethanol at the final concentration or 134.40 g/L maltodextrin 42 was added to prepare each diet. The live SN13T cell suspension (final 1–2 × 10^9^ cfu/mL) was added to the diet.

### 4.3. Ethics Statement

The animal experiment in the present study was conducted according to the “Guidelines for the Care and Use of Laboratory Animals” of Hiroshima University. All experimental procedures were approved by the “Committee of Research Facilities for Laboratory Animal Science of Hiroshima University” (Permit Numbers: A14-123 and A16-9, 19 November 2014, and 10 May 2016, respectively).

### 4.4. Animal Experiment to Evaluate Survival Rate

Male C57BL/6J specific pathogen free (SPF) mice (seven weeks of age) were purchased from Charles River Laboratories Japan, Inc. After the mice were kept for one week with a regular diet (MF, Oriental Yeast Co., Ltd., Tokyo, Japan), they were divided into four groups composed of five mice each in a plastic cage: Group I was fed only an L10015 diet without the administration of ethanol; group II was fed an L10016 diet with ethanol; group III was fed an L10016 diet with the simultaneous administration of ethanol and live SN13T cells; and group IV was fed an L10016 diet supplemented with ethanol and heat-killed SN13T cells. All mice were kept under conditions of 40%–60% humidity, 20–26 °C, and a 12 h light/12 h dark cycle. Since all of the diets contain sufficient water, the mice were not given any additional water; they had free access to the food. The diets were changed every day, and the amount of daily food intake by mice in each cage was monitored by weighing the remaining diet. The body weights of the mice were also recorded each week. The mice were distinguished by painting the tail of each a different color using felt-tip markers (Animal Marker, Muromachi Kikai Co., Ltd., Tokyo, Japan). After the rearing of mice, they were euthanized by inhalation of anesthesia with isoflurane. Feces from each cage was collected to analyze the fecal microbiota.

### 4.5. Animal Experiment to Analyze the Microbiota and Metabolites in the Intestine

**Figure 6:**
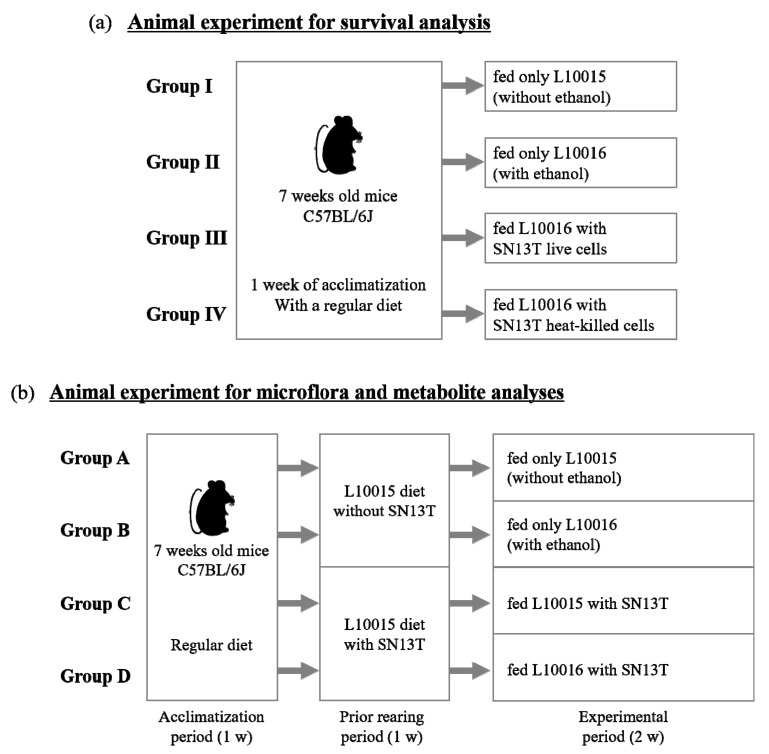
The outlines of animal experiments for survival analysis (**a**) and microbiota and metabolite analyses (**b**).

After being fed with the regular diet for one week, the male C57BL/6J SPF mice were divided into four groups: A, B, C, and D. The mice in each group were kept under the conditions described above. Prior to feeding with ethanol, the mice were kept for one week using the L10015 diet supplemented with (groups C and D) or without (groups A and B) live SN13T cells. After one week, the L10015 diet was changed to an L10016 diet in groups B and D. The diets were changed every day, and daily consumption was monitored. Two weeks after the start of the experiment, the mice were euthanized by the inhalation of anesthesia with isoflurane, and blood was collected from each mouse. The small and large intestines and the cecum were collected from each mouse to analyze the intestinal microbiota and cecal metabolites. Schemes summarizing the animal experiments are shown in Figure 6.

### 4.6. Statistical Analyses

Statistical analyses were conducted using the SPSS 17.0 software (SPSS Japan, Inc., Tokyo, Japan). The Tukey–Kramer test was applied for multiple comparisons of body weight and blood biochemical parameters. Mouse survival curves were plotted using the Kaplan–Meier method. The survival ratio between each group was analyzed using a log-rank test.

### 4.7. Analysis of the Fecal Microbiota Using the T-RFLP Method

Mouse fecal microbiota were compared according to the T-RFLP method [45] with a slight modification [46]. The unit T-RF (terminal restriction fragment) is a just expedient, and the results were indicated as bacterial taxa designated OTU (operational taxonomic unit).

### 4.8. Analysis of the Gut Microbiota via 16S rRNA Encoding Gene Sequencing

Total DNA extraction from 200–500 mg of mouse gut contents was done using a Gencheck DNA extraction kit type S/F (FASMAC Co., Ltd., Kanagawa, Japan) according to the manufacturer’s instructions. The 16S rRNA-based microbiota analysis was conducted using the Illumina MiSeq sequencing platform (Illumina Inc., San Diego, CA, USA) using a 300 bp read length paired-end protocol. The V3–V4 regions of bacterial 16S rRNA-encoding genes were PCR-amplified via TaKaRa Ex *Taq* HS (Takara Bio, Inc., Shiga, Japan) using the primers 1st_V3V4_F (5′-ACACTCTTTCCCTACACGACGCTCTTCCGATCTCCTACGGGNGGCWGCAG-3′) and 1st_V3V4_R (5′- GTGACTGGAGTTCAGACGTGTGCTCTTCCGATCTGACTACHVGGGTATCTAATCC-3′) under the following conditions: 2 min at 94 °C followed by 20 cycles of 30 s at 94 °C, 30 s at 50 °C, and 30 s at 72 °C, and finally a 5 min extension period at 72 °C. The amplified fragments were purified using an Agencourt AMPure XP (Thermo Fisher Scientific, Waltham, MA, USA) according to the manufacturer’s protocol. A 1/10 volume aliquot of the purified fragments was used for the second PCR reaction conducted with the primer set 2nd_F1 (5′-AATGATACGGCGACCACCGAGATCTACACNNNNNNNNACACTCTTTCCCTACACGACGC-3′) and 2nd_R1 (5′-CAAGCAGAAGACGGCATACGAGATNNNNNNNNGTGACTGGAGTTCAGACGTGTG-3′) using a TaKaRa Ex *Taq* HS under the following conditions (the underlined regions in each primer are index sequences designed to allow the analytical software to identify each sample): 2 min at 94 °C followed by eight cycles of 30 s at 94 °C, 30 s at 60 °C, and 30 s at 72 °C, and finally a 5 min extension period at 72 °C. The amplified fragments were also purified using an Agencourt AMPure XP. After sequencing analysis, the taxonomic assignments were done using the Quantitative Insights into Microbial Ecology (QIIME) pipeline [47].

### 4.9. Sample Preparations for Metabolite Analysis of Bowel

Metabolite analysis of the mouse bowel was performed by Human Metabolome Technologies, Inc. (Yamagata, Japan). To use liquid chromatography time-of-flight mass spectrometry (LC-TOFMS) in the analysis, a 50 mg aliquot of the bowel sample from each group was resuspended into 1 mL of methanol containing 10 μM Internal Standard Solution 1 (H3304–1002, Human Metabolome Technologies, Inc.). After centrifugation, a 200 μL aliquot of the supernatant fluid was transferred to a new tube and dried in vacuo. Each dried sample was dissolved into 50% (*v/v*) isopropanol prior to the LC-TOFMS analysis. Meanwhile, for the capillary electrophoresis (CE)-TOFMS, a 50 mg aliquot of the bowel contents in each group was resuspended into 450 μL of Milli-Q water containing 200 μM Internal Standard Solution 1. After centrifugation, a 400 μL aliquot of the supernatant fluid was filtrated using an UltrafreeMC-PLHCC 250/pk for the metabolome analysis (5000 MWCO, Human Metabolome Technologies, Inc.) to remove proteins by centrifugation at 9100× *g* for 1 h at 4 °C. An 80 μL aliquot of the filtrate was mixed with 20 μL of Milli-Q water prior to the CE-TOFMS analysis.

### 4.10. Analytical Conditions for LC-TOFMS and CE-TOFMS

To analyze metabolites contained in the bowel using LC-TOFMS, the samples were applied on an Agilent 1200 series RRLC system SL (Agilent Technologies, Inc., Santa Clara, CA, USA) equipped with an MS system Agilent LC/MSD TOF (Agilent Technologies, Inc.) using an octadecylsilyl (ODS) column (2 × 50 mm, 2 μm). For CE-TOFMS analysis, the samples were applied on an Agilent CE-TOFMS system (Agilent Technologies, Inc.) using a fused silica capillary id 50 μm × 80 mm. The analytical conditions are summarized in Table 3. The metabolites were identified by matching their times of migration and *m/z* values with the annotation table of the library (Human Metabolome Technologies, Inc.). The resultant data were used for HCA, and the analysis was done using an unweighted pair-group method of analysis (UPGMA).

## 5. Conclusions

We demonstrate in the present study that the alcohol-poisoning symptom in mice is improved by the live SN13T cell or its collaboration with other intestinal bacteria. Our study indicates that probiotic bacteria which affect the microbiota may be an attractive target to produce healthcare supplements.

## Figures and Tables

**Table 1:** The summary of changes in intestinal microbiota composition.

Part of Intestine/Alteration	Phylum	Class	Order	Family	Genus
Small intestine					
Increased ^1^		Epsilonproteobacteria *^,3^	Bacillales	Propionibacteriaceae *	Propionibacterium *
			Lactobacillales *	Paraprevotellaceae *	Staphylococcus *
			Campylobacterales *	Staphylococcaceae *	Facklamia *
				Enterococcaceae *	*Enterococcus*
				Campylobacteraceae *	Arcobacter *
					Proteus *
Decreased ^2^	Bacteroidetes *	Coriobacteriia *	Coriobacteriales *	Coriobacteriaceae *	Adlercreutzia *
	Cyanobacteria	Bacteroidiia *	Bacteroidales *	Porphyromonadaceae *	Parabacteroides *
	Proteobacteria *	Chloroplast	Streptophyta	S24-7 *	*Lactobacillus*
	TM7 *	Clostridia *	Clostridiales *	Lactobacillaceae *	*Clostridium*
	Tenericutes *	Erysipelotrichi *	Erysipelotrichales *	Clostridiaceae	Coprococcus *
	Verrucomicrobia *	Alphaproteobacteria *	Rhizobiales	Lachnospiraceae *	Allobaculum *
		Betaproteobacteria *	Burkholderiales *	Ruminococcaceae *	Sutterella *
		Deltaproteobacteria *	Desulfovibrionales *	Erysipelotrichaceae *	Bilophila *
		TM7-3 *	Spirobacillales	Methylobacteriaceae	Akkermansia *
		Mollicutes *	Pseudomonadales *	Alcaligenaceae *	
		Verrucomicrobiae *	CW040 *	Desulfovibrionaceae *	
			RF39 *	F16 *	
			Verrucomicrobiales *	Verrucomicrobiaceae *	
Cecum					
Increased		Gammaproteobacteria	Bacillales	Bifidobacteriaceae	*Bifidobacterium*
			Turicibacterales *	Staphylococcaceae *	Jeotgalicoccus *
			RF32 *	Enterococcaceae *	Staphylococcus *
			Rhodocyclales	Turicibacteraceae *	*Enterococcus*
			Enterobacteriales	Oxalobacteraceae *	Turicibacter *
				Rhodocyclaceae	Anaerotruncus *
				Enterobacteriaceae	*Anaerovorax*
					Peptoniphilus *
					Ralstonia *
					Zoogloea *
					Proteus *
Decreased	Verrucomicrobia	Verrucomicrobiae *	Rhizobiales	Prevotellaceae *	Clostridium *
			Verrucomicrobiales *	Bradyrhizobiaceae	Akkermansia *
				Verrucomicrobiaceae *	
Large intestine					
Increased		Alphaproteobacteria *	Bifidobacteriales	Bifidobacteriaceae	*Bifidobacterium*
		Gammaproteobacteria	Bacillales *	Staphylococcaceae *	*AF12*
			Turicibacterales *	Enterococcaceae *	*Jeotgalicoccus*
			Enterobacteriales	Turicibacteraceae *	Staphylococcus *
				Tissierellaceae *	Facklamia *
				Enterobacteriaceae	Enterococcus *
					Turicibacter *
					Anaerotruncus *
					*Finegoldia*
					*Enterobacter*
					Proteus *
Decreased	Verrucomicrobia	Verrucomicrobiae *	Verrucomicrobiales *	Prevotellaceae *	*Anaerofustis*
				Eubacteriaceae	Akkermansia *
				Verrucomicrobiaceae *	

The bacteria that belongs to the categories listed in the table were increased or decreased in their proportions by ethanol administration when compared with nonethanol diet control group. ^1^ “Increased” means the categories that became detectable or that increased by four times or more only after ethanol administration. ^2^ “Decreased” means the categories that became nondetectable or that decreased by four times or more only after ethanol administration. ^3^ The changed proportions were restored by administration of the live SN13T strain in the category names indicated by asterisk (*).

**Table 2:** Differences of standardized relative area in metabolites in the cecum samples isolated from each mice group.

Compound Name	Group (Ethanol/SN13T)			
	A (−/−)	B (+/−)	C (−/+)	D (+/+)
Pyruvic acid	0.133	0.913	0.372	−1.418
Propionic acid	0.141	1.139	0.017	−1.297
1,3-Diaminopropane	−0.338	1.197	0.307	−1.166
Stearoyl ethanolamide	−0.461	1.238	0.302	−1.079
5α-Pregnan-3α-ol-20-one	0.190	1.333	−0.642	−0.880
Cadaverine	0.218	1.326	−0.772	−0.772
Isobutyric acid, Butyric acid	0.018	1.397	−0.859	−0.557
*N*-Acetylglutamic acid	−0.943	1.386	−0.434	−0.009
*o*-Hydroxybenzoic acid	−0.610	1.490	−0.541	−0.339
Tyramine	−0.500	1.500	−0.500	−0.500
*N*^1^,*N*^8^-Diacetylspermidine	−0.195	1.440	−0.383	−0.861
Isovaleric acid, Valeric acid	−0.356	1.466	−0.323	−0.787
Isopropanolamine	−0.642	1.455	−0.141	−0.672
2-Hydroxy-4-methylvaleric acid	−0.748	1.367	0.126	−0.745
2-Oxoglutaric acid	−0.878	1.189	0.464	−0.775
Glycine (Gly)	0.635	−1.190	0.994	−0.439
Alanine (Ala)	0.511	−0.990	1.146	−0.667
Valine (Val)	0.683	−1.098	0.996	−0.581
Leucine (Leu)	0.573	−1.045	1.093	−0.621
Isoleucine (Ilo)	0.755	−1.053	0.948	−0.649
Methionine (Met)	0.603	−0.990	1.083	−0.696
Proline (Pro)	0.546	−1.223	1.037	−0.360
Phenylalanine (Phe)	0.566	−1.016	1.104	−0.655
Tryptophan (Try)	0.569	−0.929	1.115	−0.755
Serine (Ser)	0.602	−1.158	1.034	−0.478
Threonine (Thr)	0.520	−1.097	1.113	−0.537
Tyrosine (Tyr)	0.616	−1.068	1.056	−0.604
Cystine	1.113	−0.984	0.562	−0.691
Arginine (Arg)	0.544	−0.972	1.127	−0.699
Histidine (His)	0.570	−0.891	1.117	−0.796
Lysine (Lys)	0.530	−1.003	1.131	−0.659
Asparagine (Asn)	0.383	−0.839	1.239	−0.783
Glutamine (Gln)	0.353	−0.886	1.253	−0.720
Asparatic acid (Asp)	0.600	−1.177	1.026	−0.449
Glutamic acid (Glu)	0.701	−1.059	0.993	−0.634

**Table 3:** Summary of the analytical conditions of liquid chromatography (LC)- and capillary electrophoresis time-of-flight mass spectrometry (CE-TOFMS) analyses used in this study.

Conditions	Positive Mode	Negative Mode
LC-TOFMS conditions		
LC system	Agilent 1200 series RRLC system SL	
Column	ODS column, 2 × 50 mm, 2 μm	
MS system	Agilent LC/MSD TOF	
Column temperature	40 °C	
Mobile phase	A: H_2_O/0.1% HCOOH	
	B: Isopropano:Acetonitrile:H_2_O (63:30:5)/0.1% HCOOH, 2 mM HCOONH_4_	
Flow rate	0.3 mL/min	
Run time	20 min	
Post time	7.5 min	
Gradient condition	0–0.5 min: B 1%, 0.5–13.5 min: B 1%–100%, 13.5–20 min: B 100%	
MS ionization mode	ESI positive	ESI negative
MS nebulizer pressure	40 psi	
MS dry gas flow	10 L/min	
MS dry gas temperature	350 °C	
MS capillary voltage	3500 V	
MS scan range	*m/z* 100–1700	
Sample injection volume	1 μL	
CE-TOFMS conditions		
CE system	Agilent CE-TOFMS system	
Capillary	Fused silica capillary id 50 μm × 80 cm	
Run buffer	Cation Buffer Soln. (p/n: H3301–1001)	Anion Buffer Soln. (p/n: H3302–1021)
Rinse buffer	Cation Buffer Soln. (p/n: H3301–1001)	Anion Buffer Soln. (p/n: H3302–1021)
Sample injection	Pressure injection 50 mbar, 10 s	Pressure injection 50 mbar, 25 s
CE voltage	Positive, 27 kV	Positive, 30 kV
MS ionization	ESI positive	ESI negative
MS capillary voltage	4000 V	3500 V
MS scan range	*m/z* 50–1000	
Sheath liquid	HMT Sheath Liquid (p/n: H3301–1020)	

## Supplementary Materials

Supplementary materials can be found at https://www.mdpi.com/1422-0067/21/5/1896/s1.

## Abbreviations

ADH	alcohol dehydrogenase
ALT	alanine aminotransferase
AST	aspartate transaminase
CE	capillary electrophoresis
FAO	Food and Agriculture Organization of the United Nations
γ-GTP	γ-glutamyl transpeptidase
HCA	hierarchical cluster analysis
IBD	inflammatory bowel disease
IgA	immunoglobulin A
LAB	lactic acid bacteria
*Lb.*	*Lactobacillus*
LC	liquid chromatography
MRS	de Man, Rogosa, and Sharpe
ODS	octadecylsilyl
OTU	operational taxonomic unit
QIIME	Quantitative Insights into Microbial Ecology
rDNA	ribosomal DNA
rRNA	ribosomal RNA
SPF	specific pathogen free
TOF-MS	time-of-flight mass spectrometry
T-RFLP	terminal restriction fragment length polymorphism
UPGMA	unweighted pair-group method of analysis
