# Supplementary material for: Improvement of Alcohol-Poisoning Symptoms in Mice by the Oral Administration of Live Lactobacillus plantarum SN13T Cells
Source: Int J Mol Sci. 2020 Mar 10;21(5):1896. doi: 10.3390/ijms21051896 (PMC7084619; doi:10.3390/ijms21051896)
Supplement: Supplementary file 1 [file ijms-21-01896-s001.pdf]

Table S1.The detailed result of HCA.

Line	HMT DB †			m/z	MT/RT	Standardized Relative Area §			
	Compound name	KEGG ID	HMDB ID			Group A	Group B	Group C	Group D
1	2-Deoxyribose 1-phosphate	C00672	HMDB01351	213.015	10.65	-0.328	-0.860	-0.256	1.445
2	5-Aminovaleric acid	C00431	HMDB03355	118.087	8.96	-0.761	-0.439	-0.268	1.468
3	Citramalic acid	C02612,C02614	HMDB00426	147.030	16.95	-0.500	-0.500	-0.500	1.500
4	FA(22:2)	No ID	No ID	335.297	15.18	-0.500	-0.500	-0.500	1.500
5	Thiaproline	No ID	No ID	134.028	15.83	-0.512	-0.448	-0.539	1.499
6	FA(22:4)-1	-	-	331.264	14.52	-0.209	-0.465	-0.785	1.458
7	Ethyl arachidonate-1	-	-	350.306	14.51	-0.244	-0.280	-0.905	1.429
8	Nervonic acid-1	-	-	365.341	16.16	-0.831	0.488	-0.831	1.174
9	γ-Butyrobetaine	C01181	HMDB01161	146.118	9.01	-0.971	0.730	-0.745	0.985
10	Succinic semialdehyde	C00232	HMDB01259	101.025	9.74	-0.891	0.957	-0.836	0.770
11	XA0003	-	-	124.990	12.29	-0.847	1.100	-0.847	0.594
12	Gluconic acid	C00257	HMDB00625	195.051	8.01	-0.813	1.205	-0.832	0.441
13	Glutaric acid	C00489	HMDB00661	131.034	16.22	-0.780	1.158	-0.889	0.511
14	Gluconolactone	C00198	HMDB00150	179.056	26.73	-0.586	1.118	-1.062	0.529
15	22-Hydroxycholesterol<br>20α-Hydroxycholesterol	C05502<br>C05500	HMDB04035<br>HMDB06283	385.347	13.28	0.219	0.937	-1.416	0.260
16	Hypotaurine	C00519	HMDB00965	110.027	21.01	1.003	0.070	-1.377	0.304
17	Daminozide<br>Ala-Ala	C10996<br>C00993	No ID<br>HMDB03459	161.093	10.36	1.102	0.577	-0.986	-0.693
18	N^5-Ethylglutamine	C01047	No ID	175.108	12.78	1.102	0.590	-0.796	-0.896
19	N^8-Acetylspermidine	C01029	HMDB02189	188.176	7.02	0.823	0.908	-0.836	-0.894
20	Estrone	C00468	HMDB00145	271.168	9.70	0.786	0.943	-0.864	-0.864
21	Piperidine	C01746	No ID	86.096	7.73	0.505	1.164	-0.834	-0.834
22	4-Guanidinobutyric acid	C01035	HMDB03464	146.092	9.30	0.585	0.804	0.028	-1.418
23	Pyruvic acid	C00022	HMDB00243	87.008	12.63	0.133	0.913	0.372	-1.418
24	Propionic acid	C00163	HMDB00237	73.030	10.75	0.141	1.139	0.017	-1.297
25	1,3-Diaminopropane	C00986	HMDB00002	75.092	4.86	-0.338	1.197	0.307	-1.166
26	Stearoyl ethanolamide	No ID	No ID	328.322	14.06	-0.461	1.238	0.302	-1.079
27	α-Tocopherol acetate-1	-	-	490.425	16.10	0.061	1.353	-0.417	-0.996
28	5α-Pregnan-3α-ol-20-one	C13712	HMDB01449	319.263	11.42	0.190	1.333	-0.642	-0.880
29	Cadaverine	C01672	HMDB02322	103.123	5.50	0.218	1.326	-0.772	-0.772
30	Isobutyric acid<br>Butyric acid	C02632<br>C00246	HMDB01873<br>HMDB00039	87.045	9.73	0.018	1.397	-0.859	-0.557
31	N-Acetylglutamic acid	C00624	HMDB01138	188.056	12.87	-0.943	1.386	-0.434	-0.009
32	o-Hydroxybenzoic acid	C00805	HMDB01895	137.024	10.22	-0.610	1.490	-0.541	-0.339

Table S1.The detailed result of HCA.

Line	HMT DB †			m/z	MT/RT	Standardized Relative Area §			
	Compound name	KEGG ID	HMDB ID			Group A	Group B	Group C	Group D
33	α-Tocopherol acetate-2	-	-	490.430	16.21	-0.491	1.498	-0.564	-0.444
34	Ethyl arachidonate-2	-	-	350.306	14.69	-0.500	1.500	-0.500	-0.500
35	Tyramine	C00483	HMDB00306	138.092	9.22	-0.500	1.500	-0.500	-0.500
36	N^1,N^8-Diacetylspermidine	No ID	HMDB41947	230.186	11.49	-0.195	1.440	-0.383	-0.861
37	Isovaleric acid<br>Valeric acid	C08262<br>C00803	HMDB00718<br>HMDB00892	101.061	9.16	-0.356	1.466	-0.323	-0.787
38	Isopropanolamine	C03194,C05771	HMDB12136	76.076	7.78	-0.642	1.455	-0.141	-0.672
39	2-Hydroxy-4-methylvaleric acid	C03264	HMDB00624	131.070	8.74	-0.748	1.367	0.126	-0.745
40	2-Oxoglutaric acid	C00026	HMDB00208	145.014	20.70	-0.878	1.189	0.464	-0.775
41	3-Aminobutyric acid	No ID	No ID	104.071	8.73	-1.137	0.886	0.792	-0.542
42	Campesterol-1	-	-	383.365	16.00	-0.866	0.900	0.832	-0.866
43	*N*-Acetylglutamine	No ID	HMDB06029	187.071	7.97	-0.821	0.434	1.209	-0.821
44	Linoleyl ethanolamide-2	-	-	324.290	13.02	-0.734	0.339	1.262	-0.867
45	2-Hydroxyvaleric acid	No ID	HMDB01863	117.055	9.10	-1.215	0.140	1.218	-0.143
46	1-Methyl-4-imidazoleacetic acid	C05828	HMDB02820	141.066	9.20	-1.292	0.354	1.088	-0.151
47	Kynurenic acid	C01717	HMDB00715	190.050	6.09	-1.426	0.839	0.505	0.082
48	*N*-Methylalanine	C02721	No ID	104.071	11.51	-1.493	0.634	0.435	0.425
49	Glycocholic acid	C01921	HMDB00138	464.302	6.64	-1.488	0.367	0.670	0.451
50	Lanosterol<br>Cycloartenol	C01724<br>C01902	HMDB01251<br>No ID	409.377	16.01	-1.430	0.282	0.904	0.243
51	Pyridoxal	C00250	HMDB01545	168.067	9.80	-1.356	-0.130	0.886	0.599
52	1,2-Dipalmitoyl-glycero-3-phosphoethanolamine-2	-	-	692.527	16.09	-1.139	-0.526	0.970	0.695
53	Hyodeoxycholic acid	C15517	HMDB00733	391.286	10.81	-1.089	-0.285	1.315	0.060
54	XC0089	-	-	255.097	10.68	-0.777	-0.634	1.412	-0.001
55	Creatinine	C00791	HMDB00562	114.066	8.11	-0.747	-0.664	1.415	-0.004
56	AC(18:2)-2	-	-	424.343	10.80	-0.821	-0.692	1.350	0.163
57	FA(14:3)	No ID	No ID	221.155	12.06	-0.727	-0.771	1.363	0.135
58	Deoxycholic acid	C04483	HMDB00626	391.290	11.69	-0.581	-1.014	1.223	0.372
59	2'-Deoxyadenosine<br>5'-Deoxyadenosine	C00559<br>C05198	HMDB00101<br>HMDB01983	252.108	10.76	-0.806	-0.806	1.255	0.357
60	Asiatic acid	C08617	No ID	487.342	11.00	-0.808	-0.834	1.212	0.430
61	Ergosterol-1	-	-	397.342	15.33	-0.987	-0.689	1.108	0.568
62	5-Methylcytosine	C02376	HMDB02894	126.066	8.68	-0.847	-0.847	1.101	0.594
63	2-Hydroxyisobutyric acid	No ID	HMDB00729	103.040	9.61	-0.864	-0.864	0.954	0.773
64	1,2-Dipalmitoyl-glycero-3-phosphoethanolamine-1	-	-	692.515	16.17	-1.014	-0.635	0.503	1.146

Table S1.The detailed result of HCA.

Line	HMT DB [†]			*m/z*	MT/RT	Standardized Relative Area [§]			
	Compound name	KEGG ID	HMDB ID			Group A	Group B	Group C	Group D
65	*N*-Acetylaspartic acid	C01042	HMDB00812	174.041	14.20	-1.168	-0.374	0.377	1.164
66	Sarcosine	C00213	HMDB00271	90.055	10.57	-0.851	-0.562	0.013	1.400
67	1,2-Distearoyl-glycero-3-phosphocholine-1	-	-	790.625	16.55	-0.886	-0.672	0.255	1.303
68	Undecanoic acid	No ID	HMDB00947	185.153	7.65	-0.746	-0.802	0.224	1.323
69	Cholic acid	C00695	HMDB00619	407.280	10.70	-0.819	-0.752	0.271	1.300
70	Thiamine phosphate	C01081	HMDB02666	345.077	12.06	-0.841	-0.841	0.547	1.135
71	Cytosine	C00380	HMDB00630	112.051	8.06	-0.821	-0.884	0.638	1.067
72	AC(13:1)	No ID	No ID	356.270	9.81	-0.860	-0.860	0.715	1.004
73	Decanoic acid	C01571	HMDB00511	171.138	7.82	-0.682	-1.006	0.626	1.063
74	Isethionic acid	C05123	HMDB03903	124.992	11.38	-0.538	-1.141	0.856	0.823
75	1,2-Distearoyl-glycero-3-phosphocholine-2	-	-	790.626	16.92	-0.515	-1.149	0.951	0.714
76	*N*-Acetylgalactosamine<br>*N*-Acetylmannosamine<br>*N*-Acetylglucosamine	C01132<br>C00645<br>C00140	HMDB00853<br>HMDB01129<br>HMDB00215	222.098	25.78	-0.526	-1.137	0.982	0.681
77	Stigmasterol-2	-	-	395.368	15.93	-0.467	-1.146	1.095	0.519
78	FA(19:1)-1	-	-	295.264	14.73	-0.336	-1.251	0.973	0.613
79	Sphingomyelin(d18:1/16:0)-1	-	-	703.564	15.38	0.057	-1.406	0.910	0.439
80	3-(4-Hydroxyphenyl)propionic acid	C01744	HMDB02199	165.055	8.26	0.048	-1.410	0.885	0.477
81	XA0033	-	-	242.080	7.54	0.036	-1.422	0.783	0.603
82	Isoglutamic acid	C05574	No ID	148.061	10.21	-0.036	-1.399	0.636	0.798
83	Phosphorylcholine	C00588	HMDB01565	184.074	23.97	-0.157	-1.351	0.665	0.842
84	Cholesterol sulfate	C18043	HMDB00653	465.307	16.57	-0.320	-1.275	0.814	0.780
85	Imidazole-4-methanol	C05562	No ID	99.056	8.05	-0.478	-1.138	0.517	1.099
86	Desmosterol<br>7-Dehydrocholesterol	C01802<br>C01164	HMDB02719<br>HMDB00032	367.342	15.36	-0.171	-1.297	0.410	1.058
87	2-Hydroxyglutaric acid	C02630,C01087,C03196	HMDB00606,HMDB00694	147.029	16.22	0.382	-1.359	-0.026	1.003
88	2'-Deoxyuridine	C00526	HMDB00012	229.082	25.78	0.303	-1.464	0.367	0.794
89	*N*-Acetylglucosylamine	C01239	HMDB01104	221.112	11.12	0.382	-1.480	0.373	0.725
90	Cholesterol	C00187	HMDB00067	369.353	15.77	0.493	-1.498	0.443	0.563
91	XA0012	-	-	166.017	9.26	0.617	-1.486	0.303	0.566
92	Thymidine	C00214	HMDB00273	243.097	25.80	0.787	-1.417	0.020	0.610
93	Campesterol-2	-	-	383.368	15.92	0.856	-1.434	0.141	0.437
94	cis-11,14-Eicosadienoic acid-2	-	-	307.265	14.74	0.960	-1.406	0.232	0.214
95	cis-4,7,10,13,16,19-Docosahexaenoic acid-1	-	-	327.233	13.86	0.864	-1.441	0.365	0.212
96	5α-Cholestan-3-one-1	-	-	387.363	15.71	0.986	-1.377	0.361	0.031

Table S1.The detailed result of HCA.

Line	HMT DB †			*m/z*	MT/RT	Standardized Relative Area §			
	Compound name	KEGG ID	HMDB ID			Group A	Group B	Group C	Group D
97	Imidazole-4-acetic acid	C02835	HMDB02024	127.050	8.93	0.734	-1.475	0.439	0.302
98	2,3-Diaminopropionic acid	C03401,C06393	HMDB02006	105.066	7.92	0.602	-1.495	0.499	0.394
99	2-Hydroxybutyric acid	C05984	HMDB00008	103.040	9.60	0.581	-1.492	0.557	0.354
100	2'-Deoxycytidine	C00881	HMDB00014	228.099	10.54	0.694	-1.471	0.552	0.224
101	*N*-Acetylserine	No ID	HMDB02931	148.061	27.11	0.643	-1.466	0.635	0.188
102	*N*-Acetylalanine	No ID	HMDB00766	130.051	8.75	0.557	-1.480	0.653	0.270
103	Dihydroxyacetone phosphate	C00111	HMDB01473	168.991	12.36	0.542	-1.475	0.686	0.247
104	Taurine	C00245	HMDB00251	126.022	25.75	0.370	-1.460	0.806	0.284
105	1-Pyrroline 5-carboxylic acid	C03912	HMDB01301	114.056	12.45	0.472	-1.389	0.921	-0.003
106	Uric acid	C00366	HMDB00289	167.021	8.77	0.521	-1.414	0.854	0.039
107	*cis*-4-Hydroxyproline	C03440	HMDB06055	132.065	12.45	0.540	-1.433	0.805	0.089
108	Creatine	C00300	HMDB00064	132.077	9.88	0.545	-1.433	0.800	0.088
109	1-Methyladenosine	C02494	HMDB03331	282.122	11.13	0.767	-1.390	0.690	-0.067
110	Carnitine	C00318,C00487,C15025	HMDB00062	162.113	9.49	0.737	-1.359	0.767	-0.145
111	Glucose 1-phosphate	C00103	HMDB01586	259.022	9.94	0.694	-1.369	0.793	-0.118
112	CMP-*N*-acetylneuraminate	C00128	HMDB01176	613.143	8.03	0.699	-1.352	0.811	-0.158
113	N^6-Methyladenine	C08434	HMDB02099	150.078	8.83	0.694	-1.352	0.816	-0.159
114	5-Hydroxylysine	C16741	HMDB00450	163.109	7.93	0.346	-1.334	1.047	-0.059
115	SDMA	No ID	HMDB03334	203.151	8.68	0.256	-1.366	1.031	0.079
116	Betaine	C00719	HMDB00043	118.087	12.66	0.168	-1.376	1.022	0.187
117	Prostaglandin D1<br>Prostaglandin E1	C06438<br>C04741	HMDB05102<br>HMDB01442	353.232	9.79	0.112	-1.392	0.979	0.301
118	FA(17:0)-1<br>Heptadecanoic acid-1	-<br>-	-<br>-	269.249	14.46	-0.111	-1.258	1.168	0.201
119	Tauroursodeoxycholic acid	No ID	HMDB00874	498.288	11.35	-0.219	-1.178	1.241	0.156
120	Adenine	C00147	HMDB00034	136.063	8.48	-0.254	-1.064	1.343	-0.026
121	AC(18:2)-3	-	-	424.343	11.07	-0.277	-1.057	1.344	-0.011
122	Taurocholic acid	C05122	HMDB00036	514.281	11.50	-0.486	-0.988	1.329	0.144
123	Taurodeoxycholic acid	C05463	HMDB00896	498.289	12.62	-0.408	-0.993	1.359	0.042
124	FA(22:3)-1	-	-	333.279	14.72	-0.369	-0.903	1.423	-0.152
125	cis-5,8,11,14,17-Eicosapentaenoic acid<br>Abietic acid	C06428<br>C06087	HMDB01999<br>No ID	301.218	13.39	-0.306	-0.922	1.421	-0.192
126	*N*-Acetylneuraminic acid	C00270	HMDB00230	308.097	7.11	-0.361	-0.851	1.446	-0.233
127	β-Ala	C00099	HMDB00056	90.055	8.14	-0.437	-0.781	1.463	-0.245
128	Lactic acid	C00186,C00256,C01432	HMDB00190,HMDB01311	89.024	10.54	-0.261	-0.861	1.444	-0.322

Table S1.The detailed result of HCA.

Line	HMT DB †			*m/z*	MT/RT	Standardized Relative Area §			
	Compound name	KEGG ID	HMDB ID			Group A	Group B	Group C	Group D
129	Sphinganine	C00836	HMDB00269	302.307	10.86	-0.205	-0.855	1.442	-0.383
130	Riboflavin	C00255	HMDB00244	377.146	6.04	-0.129	-0.874	1.428	-0.425
131	1-Palmitoyl-glycero-3-phosphocholine-1	-	-	496.340	12.61	-0.280	-0.643	1.482	-0.559
132	1-Oleoyl-glycero-3-phosphocholine-2	-	-	522.358	12.93	-0.297	-0.625	1.485	-0.562
133	1-Myristoyl-glycero-3-phosphocholine-1	-	-	468.309	11.98	-0.326	-0.632	1.488	-0.530
134	Alloisoleucine	No ID	HMDB00557	132.102	11.73	-0.466	-0.621	1.494	-0.406
135	Lithocholic acid	C03990	HMDB00761	375.290	12.49	-0.507	-0.529	1.499	-0.463
136	*p*-Hydroxybenzoic acid	C00156	HMDB00500	137.024	9.26	-0.500	-0.500	1.500	-0.500
137	CMP	C00055	HMDB00095	322.047	9.42	-0.500	-0.500	1.500	-0.500
138	Glycerophosphocholine	C00670	HMDB00086	258.111	25.22	-0.500	-0.500	1.500	-0.500
139	15(S)-HETE	C04742	HMDB03876	319.227	12.32	-0.500	-0.500	1.500	-0.500
140	AC(17:0)	No ID	No ID	414.358	11.45	-0.500	-0.500	1.500	-0.500
141	1-Myristoyl-glycero-3-phosphocholine-2	-	-	468.310	11.83	-0.500	-0.500	1.500	-0.500
142	Hecogenin	C08902	No ID	431.320	11.89	-0.500	-0.500	1.500	-0.500
143	Etiocholan-3α-ol-17-one glucuronide	C11136	HMDB04484	465.258	10.14	-0.500	-0.500	1.500	-0.500
144	FA(16:2)	No ID	No ID	251.203	13.21	-0.500	-0.500	1.500	-0.500
145	3'-AMP	C01367	HMDB03540	346.053	9.51	-0.500	-0.500	1.500	-0.500
146	β-Estradiol<br>17α-Estradiol	C00951<br>C02537	HMDB00151<br>HMDB00429	273.185	9.70	-0.500	-0.500	1.500	-0.500
147	Taurochenodeoxycholic acid	C05465	HMDB00951	498.300	12.32	-0.500	-0.500	1.500	-0.500
148	Taurolithocholic acid	C02592	HMDB00722	482.293	13.27	-0.500	-0.500	1.500	-0.500
149	β-Ala-Lys	C05341	No ID	218.151	7.52	-0.500	-0.500	1.500	-0.500
150	GMP	C00144	HMDB01397	362.050	9.02	-0.500	-0.500	1.500	-0.500
151	*p*-Hydroxyphenylpyruvic acid	C01179	HMDB00707	179.034	8.64	-0.500	-0.500	1.500	-0.500
152	Phytosphingosine	C12144	HMDB04610	318.302	10.61	-0.528	-0.483	1.500	-0.489
153	1-Oleoyl-glycero-3-phosphocholine-1	-	-	522.356	12.78	-0.566	-0.378	1.495	-0.551
154	Glucuronic acid-2<br>Galacturonic acid-2	-<br>-	-<br>-	193.034	7.97	-0.514	-0.368	1.493	-0.610
155	Glu-Glu	C01425	No ID	277.104	12.22	-0.614	-0.284	1.483	-0.585
156	Glucuronic acid-1<br>Galacturonic acid-1	-<br>-	-<br>-	193.035	8.11	-0.560	-0.170	1.458	-0.728
157	Ser-Glu	No ID	No ID	235.093	11.63	-0.453	-0.208	1.456	-0.795
158	His-Glu	No ID	No ID	285.121	8.42	-0.291	-0.443	1.474	-0.739
159	Glycerol	C00116	HMDB00131	93.055	25.76	-0.244	-0.374	1.451	-0.833
160	Thiamine	C00378	HMDB00235	265.113	7.32	-0.168	-0.357	1.426	-0.901

Table S1.The detailed result of HCA.

Line	HMT DB †			m/z	MT/RT	Standardized Relative Area §			
	Compound name	KEGG ID	HMDB ID			Group A	Group B	Group C	Group D
161	γ-Tocopherol	C02483	No ID	417.373	15.76	-0.132	-0.349	1.412	-0.931
162	2-Oxoisovaleric acid	C00141	HMDB00019	115.040	10.00	-0.040	-0.455	1.403	-0.908
163	4-Methyl-2-oxovaleric acid<br>3-Methyl-2-oxovaleric acid	C00233<br>C00671,C03465	HMDB00695<br>HMDB00491	129.055	9.38	-0.011	-0.564	1.409	-0.835
164	Ricinoleic acid-1	-	-	297.244	12.34	-0.010	-0.705	1.418	-0.702
165	*O*-Acetylcarnitine	C02571	HMDB00201	204.124	10.06	-0.058	-0.669	1.432	-0.705
166	dAMP	C00360	HMDB00905	330.060	9.22	-0.057	-0.688	1.432	-0.688
167	Myristic acid	C06424	HMDB00806	227.202	13.50	-0.066	-0.692	1.435	-0.677
168	1-Hexadecyl-2-acetyl-glycero-3-phosphocholine-1	-	-	524.363	12.92	-0.187	-0.609	1.465	-0.669
169	Palmitoleic acid	C08362	HMDB03229	253.218	13.66	-0.128	-0.679	1.452	-0.645
170	1-Palmitoyl-glycero-3-phosphocholine-2	-	-	496.345	12.77	-0.065	-0.771	1.431	-0.594
171	Indole-3-carboxaldehyde	C08493	HMDB29737	146.061	6.94	-0.069	-0.821	1.426	-0.536
172	1-Hexadecyl-2-acetyl-glycero-3-phosphocholine-3<br>1-Stearoyl-glycero-3-phosphocholine-2	-<br>-	-<br>-	524.373	13.35	-0.096	-0.832	1.430	-0.503
173	1-Stearoyl-glycero-3-phosphocholine-1	-	-	524.376	13.50	-0.010	-0.855	1.406	-0.541
174	Glycerol 3-phosphate	C00093	HMDB00126	171.006	11.84	0.013	-0.849	1.400	-0.564
175	FA(20:3)-2	-	-	305.249	14.22	0.047	-0.780	1.396	-0.663
176	Linolenic acid	C06427	HMDB01388	277.217	13.49	0.147	-0.869	1.352	-0.630
177	FA(22:4)-2	-	-	331.265	14.30	0.132	-0.824	1.362	-0.670
178	Sphingosine	C00319	HMDB00252	300.292	10.68	0.108	-0.768	1.374	-0.714
179	FA(24:5)	No ID	No ID	357.280	14.50	0.128	-0.747	1.366	-0.747
180	Palmitoylethanolamide-1	-	-	300.289	13.45	0.139	-0.750	1.362	-0.750
181	Tricosanoic acid-2	-	-	353.341	16.04	0.145	-0.752	1.359	-0.752
182	FA(19:0)-1	-	-	297.279	15.10	0.171	-0.759	1.348	-0.759
183	Adenosine	C00212	HMDB00050	268.106	11.03	0.184	-0.763	1.342	-0.763
184	AMP	C00020	HMDB00045	346.054	9.15	0.185	-0.763	1.342	-0.763
185	FA(19:1)-2	-	-	295.266	14.64	0.188	-0.764	1.341	-0.764
186	Linoleic acid	C01595	HMDB00673	279.235	13.86	0.233	-0.783	1.320	-0.769
187	*N*-Glycolylneuraminic acid	C03410	HMDB00833	324.092	7.09	0.242	-0.890	1.309	-0.661
188	Hypoxanthine	C00262	HMDB00157	137.046	12.37	0.244	-0.949	1.299	-0.594
189	19-Methylarachidic acid-1<br>Heneicosanoic acid-1	-<br>-	-<br>-	325.311	15.68	0.325	-0.956	1.259	-0.629
190	Citrulline	C00327	HMDB00904	176.104	12.40	0.364	-0.997	1.231	-0.598
191	dCMP	C00239	HMDB01202	306.049	9.57	0.329	-1.020	1.243	-0.553
192	FA(26:0)	No ID	No ID	395.388	16.74	0.313	-0.994	1.257	-0.577

Table S1.The detailed result of HCA.

Line	HMT DB †			m/z	MT/RT	Standardized Relative Area §			
	Compound name	KEGG ID	HMDB ID			Group A	Group B	Group C	Group D
193	dTMP	C00364	HMDB01227	321.049	9.39	0.302	-0.988	1.264	-0.578
194	cis-11,14-Eicosadienoic acid-1	-	-	307.264	14.49	0.250	-1.005	1.284	-0.529
195	Uracil	C00106	HMDB00300	113.035	25.76	0.250	-1.020	1.279	-0.509
196	XA0004	-	-	144.031	9.17	0.199	-1.029	1.297	-0.467
197	AC(18:0)	No ID	No ID	428.373	11.92	0.180	-1.010	1.311	-0.480
198	cis-4,7,10,13,16,19-Docosahexaenoic acid-2	-	-	327.234	13.70	0.143	-1.018	1.321	-0.445
199	FA(17:0)-2<br>Heptadecanoic acid-2	-<br>-	-<br>-	269.249	14.56	0.164	-0.945	1.333	-0.551
200	FA(17:1)	No ID	No ID	267.233	14.00	0.127	-0.972	1.340	-0.494
201	Azelaic acid	C08261	HMDB00784	187.097	11.83	0.032	-0.985	1.364	-0.411
202	FA(22:5)-1	-	-	329.248	13.91	-0.076	-0.989	1.384	-0.319
203	Glycodeoxycholic acid	C05464	HMDB00631	448.305	11.08	-0.006	-1.103	1.319	-0.209
204	*myo* -Inositol 1-phosphate<br>*myo* -Inositol 3-phosphate	C01177<br>C04006	HMDB00213<br>HMDB06814	259.021	10.07	0.147	-1.223	1.211	-0.135
205	1-Palmitoyl-glycero-3-phosphoethanolamine-1	-	-	452.277	12.72	0.218	-1.186	1.218	-0.250
206	Xanthine	C00385	HMDB00292	153.041	22.47	0.235	-1.185	1.213	-0.262
207	Ursodeoxycholic acid	C07880	HMDB00946	391.284	10.70	0.187	-1.162	1.242	-0.267
208	Cytidine	C00475	HMDB00089	244.093	10.82	0.227	-1.132	1.246	-0.340
209	N^6-Methyllysine	C02728	HMDB02038	161.130	7.88	0.353	-1.263	1.113	-0.203
210	XC0040	-	-	174.087	13.74	0.421	-1.259	1.085	-0.247
211	GABA	C00334	HMDB00112	104.071	8.55	0.460	-1.271	1.055	-0.243
212	Choline	C00114	HMDB00097	104.107	7.58	0.439	-1.227	1.098	-0.310
213	Betaine aldehyde_+H_2O	C00576	HMDB01252	120.102	8.29	0.479	-1.249	1.061	-0.291
214	Diethanolamine	C06772	HMDB04437	106.087	8.51	0.494	-1.242	1.057	-0.309
215	Pentadecanoic acid	No ID	HMDB00826	241.217	13.88	0.521	-1.237	1.045	-0.328
216	*S* -Sulfocysteine	C05824	HMDB00731	199.969	10.86	0.504	-1.219	1.066	-0.351
217	Pro	C00148,C00763,C16435	HMDB00162,HMDB03411	116.071	12.13	0.546	-1.223	1.037	-0.360
218	N^6-Acetyllysine	C02727	HMDB00206	189.123	12.86	0.589	-1.247	0.993	-0.334
219	XA0019	-	-	191.019	8.16	0.564	-1.311	0.958	-0.211
220	Ergosterol-2	-	-	397.345	15.51	0.643	-1.312	0.901	-0.231
221	ADMA	C03626	HMDB01539	203.150	8.52	0.696	-1.252	0.908	-0.352
222	Urocanic acid	C00785	HMDB00301	139.051	9.21	0.690	-1.250	0.914	-0.353
223	Mucic acid	C00879,C01807	HMDB00639	209.029	13.70	0.661	-1.218	0.959	-0.401
224	Gly	C00037	HMDB00123	76.040	9.23	0.635	-1.190	0.994	-0.439
225	Asp	C00049,C00402,C16433	HMDB00191,HMDB06483	134.045	12.97	0.600	-1.177	1.026	-0.449

Table S1.The detailed result of HCA.

Line	HMT DB †			m/z	MT/RT	Standardized Relative Area §			
	Compound name	KEGG ID	HMDB ID			Group A	Group B	Group C	Group D
226	Ser	C00065,C00716,C00740	HMDB00187,HMDB03406	106.050	11.14	0.602	-1.158	1.034	-0.478
227	2-Aminoisobutyric acid<br>2-Aminobutyric acid	C03665<br>C02261,C02356	HMDB01906<br>HMDB00452	104.071	10.78	0.606	-1.159	1.031	-0.478
228	Ribulose 5-phosphate	C00199,C01101	HMDB00618	229.011	10.78	0.674	-1.159	0.979	-0.493
229	*N*-Acetylmethionine	C02712	HMDB11745	190.054	8.00	0.636	-1.142	1.016	-0.510
230	7-Methylguanine	C02242	HMDB00897	166.073	9.16	0.587	-1.131	1.056	-0.512
231	Val	C00183,C06417,C16436	HMDB00883	118.087	11.21	0.683	-1.098	0.996	-0.581
232	5-Oxoproline	C01879	HMDB00267	128.035	9.32	0.735	-1.129	0.941	-0.548
233	Uridine	C00299	HMDB00296	245.078	25.83	0.743	-1.180	0.911	-0.474
234	N_{ω}-Methylarginine	No ID	No ID	189.134	8.29	0.868	-1.182	0.790	-0.475
235	Sphingomyelin(d18:1/18:0)	No ID	HMDB01348	731.603	16.17	0.873	-1.136	0.807	-0.544
236	XC0132	-	-	325.162	9.68	0.915	-1.097	0.778	-0.595
237	2'-Deoxyguanosine	C00330	HMDB00085	268.105	12.89	0.966	-1.115	0.712	-0.562
238	Guanosine	C00387	HMDB00133	284.099	14.06	0.915	-1.016	0.800	-0.700
239	Inosine	C00294	HMDB00195	269.089	22.56	0.833	-1.086	0.867	-0.613
240	Pantothenic acid	C00864	HMDB00210	218.103	7.55	0.777	-1.081	0.921	-0.617
241	Ile	C00407,C06418,C16434	HMDB00172	132.102	11.44	0.755	-1.053	0.948	-0.649
242	Fumaric acid	C00122	HMDB00134	115.003	24.40	0.798	-1.015	0.917	-0.701
243	Malic acid	C00149,C00497,C00711	HMDB00156,HMDB00744	133.014	20.56	0.777	-0.981	0.943	-0.739
244	Cystathionine	C00542,C02291	HMDB00099	223.076	11.07	0.901	-0.833	0.830	-0.898
245	XC0017	-	-	130.097	8.71	0.859	-0.866	0.873	-0.866
246	AC(16:1)	No ID	No ID	398.326	10.72	0.848	-0.866	0.884	-0.866
247	Erucic acid	C08316	HMDB02068	337.311	15.43	0.854	-0.895	0.877	-0.836
248	Linoleyl ethanolamide-1	-	-	324.282	12.84	0.826	-0.866	0.906	-0.866
249	*N*-Acetylglycine	No ID	HMDB00532	116.035	9.39	0.815	-0.865	0.915	-0.865
250	Arachidic acid	C06425	HMDB02212	311.296	15.44	0.730	-0.893	0.992	-0.828
251	cis-11-Eicosenoic acid	C16526	HMDB02231	309.281	14.92	0.721	-0.877	1.000	-0.844
252	4-Pyridoxic acid	C00847	HMDB00017	182.045	8.65	0.693	-0.858	1.023	-0.858
253	XC0065	-	-	221.093	14.82	0.689	-0.858	1.027	-0.858
254	Oxypurinol	C07599	HMDB00786	153.041	25.53	0.666	-0.856	1.045	-0.856
255	Gly-Asp	No ID	No ID	191.067	11.06	0.683	-0.835	1.031	-0.879
256	Behenic acid	C08281	HMDB00944	339.327	15.91	0.642	-0.835	1.064	-0.871
257	5-Methyl-2'-deoxycytidine	C03592	HMDB02224	242.112	10.92	0.642	-0.853	1.064	-0.853
258	Palmitoylcarnitine	C02990	HMDB00222	400.342	11.22	0.644	-0.889	1.062	-0.817
259	Gly-Gly	C02037	HMDB11733	133.062	9.32	0.676	-0.887	1.036	-0.826

Table S1.The detailed result of HCA.

Line	HMT DB †			*m/z*	MT/RT	Standardized Relative Area §			
	Compound name	KEGG ID	HMDB ID			Group A	Group B	Group C	Group D
260	FA(15:0)	No ID	No ID	241.218	13.74	0.695	-1.014	1.007	-0.688
261	Glu	C00025,C00217,C00302	HMDB00148,HMDB03339	148.061	12.25	0.701	-1.059	0.993	-0.634
262	1-Hexadecyl-2-acetyl-glycero-3-phosphocholine-2	-	-	524.375	13.04	0.690	-1.055	1.003	-0.637
263	Argininosuccinic acid	C03406	HMDB00052	291.130	10.52	0.641	-1.088	1.031	-0.584
264	Ornithine	C00077,C00515,C01602	HMDB00214,HMDB03374	133.097	7.54	0.604	-1.082	1.060	-0.583
265	Tyr	C00082,C01536,C06420	HMDB00158	182.082	12.76	0.616	-1.068	1.056	-0.604
266	Ethanolamine	C00189	HMDB00149	62.060	7.02	0.631	-1.061	1.047	-0.616
267	Hydroxyproline	C01157	HMDB00725	132.066	13.56	0.649	-1.005	1.045	-0.690
268	1-Myristoyl-glycero-3-phosphoethanolamine	No ID	HMDB11500	424.246	12.06	0.644	-1.033	1.044	-0.654
269	Carnosine	C00386	HMDB00033	227.115	7.47	0.612	-1.037	1.067	-0.642
270	Leu	C00123,C01570,C16439	HMDB00687	132.102	11.56	0.573	-1.045	1.093	-0.621
271	Phe	C00079,C02057,C02265	HMDB00159	166.087	12.45	0.566	-1.016	1.104	-0.655
272	Met	C00073,C00855,C01733	HMDB00696	150.059	12.03	0.603	-0.990	1.083	-0.696
273	Stearic acid	C01530	HMDB00827	283.266	14.87	0.585	-0.986	1.096	-0.695
274	Trp	C00078,C00525,C00806	HMDB00929	205.098	12.36	0.569	-0.929	1.115	-0.755
275	FA(25:0)	No ID	No ID	381.373	16.53	0.576	-0.924	1.111	-0.762
276	His	C00135,C00768,C06419	HMDB00177	156.077	8.09	0.570	-0.891	1.117	-0.796
277	Tricosanoic acid-1	-	-	353.342	16.13	0.525	-0.890	1.149	-0.783
278	Palmitic acid	C00249	HMDB00220	255.235	14.23	0.479	-0.883	1.179	-0.775
279	1-Palmitoyl-glycero-3-phosphoethanolamine-2	-	-	452.278	12.85	0.522	-0.954	1.144	-0.713
280	cis-8,11,14-Eicosatrienoic acid	C03242	HMDB02925	305.249	14.07	0.559	-0.957	1.119	-0.721
281	Arg	C00062,C00792	HMDB00517,HMDB03416	175.119	7.89	0.544	-0.972	1.127	-0.699
282	Lys	C00047,C00739,C16440	HMDB00182,HMDB03405	147.113	7.60	0.530	-1.003	1.131	-0.659
283	Ala	C00041,C00133,C01401	HMDB00161,HMDB01310	90.055	10.05	0.511	-0.990	1.146	-0.667
284	19-Methylarachidic acid-2<br>Heneicosanoic acid-2	-<br>-	-<br>-	325.311	15.61	0.467	-0.982	1.176	-0.661
285	1-Methylhistidine<br>3-Methylhistidine	No ID<br>C01152	HMDB00001<br>HMDB00479	170.092	8.32	0.415	-0.986	1.206	-0.635
286	Thr	C00188,C00820	HMDB00167	120.066	11.77	0.520	-1.097	1.113	-0.537
287	Methionine sulfoxide	C02989	HMDB02005	166.054	13.24	0.500	-1.115	1.119	-0.504
288	Glyceric acid	C00258	HMDB00139,HMDB06372	105.019	10.17	0.370	-1.094	1.200	-0.476
289	N^6,N^6,N^6-Trimethyllysine	C03793	HMDB01325	189.160	7.97	0.419	-1.076	1.180	-0.523
290	Gln	C00064,C00303,C00819	HMDB00641,HMDB03423	147.077	12.05	0.353	-0.886	1.253	-0.720
291	Arachidonic acid	C00219	HMDB01043	303.236	13.79	0.354	-0.885	1.253	-0.721
292	FA(22:5)-2	-	-	329.249	14.09	0.380	-0.885	1.238	-0.734

Table S1.The detailed result of HCA.

Line	HMT DB †			m/z	MT/RT	Standardized Relative Area §			
	Compound name	KEGG ID	HMDB ID			Group A	Group B	Group C	Group D
293	Asn	C00152,C01905,C16438	HMDB00168	133.061	11.72	0.383	-0.839	1.239	-0.783
294	FA(19:0)-2	-	-	297.280	15.17	0.398	-0.814	1.231	-0.814
295	AC(14:0)	No ID	No ID	372.310	10.50	0.386	-0.812	1.238	-0.812
296	Tyr-Glu	No ID	No ID	311.123	12.48	0.378	-0.810	1.242	-0.810
297	FA(24:4)	No ID	No ID	359.294	14.80	0.365	-0.808	1.250	-0.808
298	FA(24:0)	No ID	No ID	367.358	16.33	0.292	-0.793	1.290	-0.789
299	Oleoyl ethanolamide-2	-	-	326.309	13.61	0.316	-0.797	1.277	-0.797
300	2,6-Diaminopimelic acid	C00666	HMDB01370	191.104	9.88	0.415	-0.799	1.221	-0.836
301	Nervonic acid-2	-	-	365.342	15.88	0.450	-0.792	1.199	-0.857
302	Oleic acid	C00712	HMDB00207	281.252	14.33	0.434	-0.846	1.209	-0.797
303	AC(20:1)	No ID	No ID	454.389	12.01	0.460	-0.846	1.192	-0.807
304	Trimethylamine *N*-oxide	C01104	HMDB00925	76.076	7.30	0.470	-0.828	1.186	-0.828
305	AC(20:0)	No ID	No ID	456.403	12.59	0.478	-0.830	1.181	-0.830
306	AC(18:1)-2	-	-	426.359	11.50	0.482	-0.830	1.179	-0.830
307	Biotin	C00120	HMDB00030	245.095	5.79	0.515	-0.836	1.156	-0.836
308	Thr-Asp	No ID	No ID	235.094	11.91	0.522	-0.799	1.151	-0.874
309	Pyridoxamine	C00534	HMDB01431	169.098	6.58	0.375	-0.711	1.240	-0.904
310	*p*-Hydroxyphenylacetic acid	C00642	HMDB00020	151.039	8.57	0.428	-0.471	1.160	-1.118
311	Dyphylline	C07819	No ID	255.108	25.79	0.274	-0.365	1.226	-1.135
312	AC(18:2)-1	-	-	424.343	10.96	0.504	-0.329	1.057	-1.232
313	*N*-Acetyllysine	C12989	HMDB00446	189.123	10.95	0.559	-0.146	0.929	-1.343
314	*N*-Acetylasparagine	No ID	HMDB06028	173.055	8.33	0.634	-0.136	0.859	-1.357
315	XC0001	-	-	72.081	7.07	0.473	0.085	0.864	-1.422
316	*N*-Methylproline	No ID	No ID	130.087	14.06	0.343	0.329	0.792	-1.465
317	*O*-Acetylhomoserine<br>2-Aminoadipic acid	C01077<br>C00956	No ID<br>HMDB00510	162.077	12.30	0.286	0.065	1.019	-1.370
318	Tartaric acid	C00898	HMDB00956	149.008	21.53	0.102	0.150	1.086	-1.337
319	*N*-Acetylornithine	C00437	HMDB03357	175.108	10.56	-0.009	-0.117	1.284	-1.157
320	Oleoyl ethanolamide-1	-	-	326.306	13.50	-0.025	-0.223	1.332	-1.084
321	*N*-Acetylhistidine	C02997	No ID	198.088	10.99	-0.222	-0.054	1.344	-1.068
322	5α-Cholestan-3-one-2	-	-	387.362	15.94	-0.383	0.081	1.335	-1.032
323	Arg-Glu	No ID	No ID	304.164	8.35	-0.158	0.148	1.220	-1.210
324	Palmitoylethanolamide-2	-	-	300.290	13.35	-0.115	0.134	1.209	-1.228
325	*N*-Acetylmuramic acid	C02713	No ID	292.103	7.18	-0.311	0.595	0.978	-1.262
326	5α-Cholestan-3-one-3	-	-	387.364	15.83	-0.055	0.467	0.955	-1.367

Table S1.The detailed result of HCA.

Line	HMT DB †			m/z	MT/RT	Standardized Relative Area §			
	Compound name	KEGG ID	HMDB ID			Group A	Group B	Group C	Group D
327	Succinic acid	C00042	HMDB00254	117.019	20.30	-0.041	0.468	0.946	-1.373
328	Hydroxyindole	C02040	No ID	134.060	25.79	0.500	0.541	0.458	-1.499
329	Hexanoic acid	C01585	HMDB00535	115.076	8.77	0.673	0.330	0.482	-1.485
330	6-Hydroxyhexanoic acid	C06103	No ID	131.071	8.47	0.819	0.188	0.442	-1.449
331	Spermidine	C00315	HMDB01257	146.165	4.99	1.199	-0.139	0.170	-1.231
332	α-Tocopherol	C02477	HMDB01893	431.389	15.96	1.041	-0.060	0.356	-1.337
333	Sitosterol-1	-	-	397.380	15.77	1.046	-0.224	0.461	-1.282
334	N^2-Acetylaminoadipic acid	C12986	No ID	202.070	12.10	0.957	-0.215	0.568	-1.310
335	Homoserine	C00263	HMDB00719	120.066	11.28	0.889	-0.300	0.691	-1.281
336	Indole-3-acetic acid	C00954	HMDB00197	174.054	8.42	0.937	-0.408	0.688	-1.218
337	Galactosamine<br>Glucosamine	C02262<br>C00329	No ID<br>HMDB01514	180.087	10.35	0.918	-0.532	0.756	-1.142
338	Trimethylamine	C00565	HMDB00906	60.082	6.49	1.190	-0.724	0.456	-0.922
339	Stigmasterol-1	-	-	395.369	15.77	1.233	-0.883	0.391	-0.740
340	FA(20:3)-1	-	-	305.249	14.42	1.168	-0.914	0.493	-0.747
341	Ethanolamine phosphate	C00346	HMDB00224	140.011	7.90	1.168	-0.833	0.499	-0.833
342	AC(18:1)-1	-	-	426.358	11.38	1.128	-0.864	0.557	-0.820
343	N-Acetylsphingosine	C00195	No ID	342.301	13.05	1.073	-0.852	0.630	-0.852
344	10-Hydroxydecanoic acid	C02774	No ID	187.135	7.66	1.041	-0.856	0.671	-0.856
345	Nicotinic acid	C00253	HMDB01488	124.039	11.17	1.035	-0.904	0.677	-0.808
346	Citric acid	C00158	HMDB00094	191.019	25.34	1.044	-0.981	0.656	-0.720
347	Cystine	C00491,C01420	HMDB00192	241.032	12.33	1.113	-0.984	0.562	-0.691
348	2-Oxobutyric acid	C00109	HMDB00005	101.024	10.95	1.001	-1.290	0.527	-0.237
349	Sphingomyelin(d18:1/16:0)-2	-	-	703.583	15.75	0.949	-1.347	0.520	-0.122
350	N^6-Methyl-2'-deoxyadenosine	C03795	No ID	266.126	11.15	0.895	-1.324	0.635	-0.207
351	Carboxymethyllysine	No ID	No ID	205.117	10.27	1.107	-1.183	0.470	-0.394
352	Flavanone	C00766	No ID	225.092	10.44	1.148	-1.182	0.394	-0.360
353	FA(22:3)-2	-	-	333.279	14.99	1.253	-1.056	0.286	-0.483
354	Formiminoglutamic acid	No ID	HMDB00854	173.056	8.03	1.289	-1.040	0.211	-0.461
355	Isovalerylalanine<br>N-Acetylleucine	No ID<br>C02710	HMDB00747<br>HMDB11756	172.098	7.87	1.323	-1.037	0.117	-0.403
356	Xanthosine	C01762	HMDB00299	283.066	7.52	1.334	-1.064	0.026	-0.296
357	cis-13-Eicosenoic acid	No ID	No ID	309.280	15.03	1.360	-1.049	-0.117	-0.194
358	3-Hydroxybutyric acid	C01089,C03197	HMDB00011,HMDB00357,HMDB00442	103.040	9.36	1.423	-0.855	-0.079	-0.489
359	Morpholine	C14452	HMDB31581	88.077	7.37	1.326	-0.772	0.219	-0.772

Table S1.The detailed result of HCA.

Line	HMT DB †			*m/z*	MT/RT	Standardized Relative Area §			
	Compound name	KEGG ID	HMDB ID			Group A	Group B	Group C	Group D
360	6-Aminohexanoic acid	C02378	HMDB01901	132.103	9.33	1.396	-0.670	0.048	-0.775
361	Ricinoleic acid-2	-	-	297.244	12.43	1.470	-0.630	-0.210	-0.630
362	*N*-Acetylputrescine	C02714	HMDB02064	131.118	9.43	1.449	-0.496	-0.158	-0.794
363	4-Methyl-5-thiazoleethanol	C04294	No ID	144.048	8.93	1.370	-0.114	-0.229	-1.028
364	Putrescine	C00134	HMDB01414	89.108	5.19	1.393	-0.008	-0.467	-0.918
365	*p*-Aminobenzoic acid	C00568	HMDB01392	138.055	10.78	1.215	0.303	-0.385	-1.133
366	N^2-Succinylornithine	No ID	HMDB01199	233.114	11.53	1.460	-0.165	-0.668	-0.628
367	2-Aminoethylphosphonic acid	C03557	HMDB11747	124.017	7.73	1.500	-0.500	-0.500	-0.500
368	8-Hydroxyoctanoic acid<br>2-Hydroxyoctanoic acid	No ID<br>No ID	No ID<br>HMDB00711	159.103	7.99	1.500	-0.500	-0.500	-0.500
369	Anthranilic acid	C00108	HMDB01123	138.055	11.84	1.500	-0.500	-0.500	-0.500
370	Glycolic acid	C00160	HMDB00115	75.009	12.35	1.500	-0.500	-0.500	-0.500
371	Sitosterol-2	-	-	397.384	16.08	1.447	-0.589	-0.738	-0.120
372	*N*-Methylglutamic acid	C01046	No ID	162.076	14.28	1.436	-0.683	-0.683	-0.070
373	Sedoheptulose 7-phosphate	C05382	HMDB01068	289.033	9.43	1.296	-1.093	-0.331	0.128
374	Glucose 6-phosphate	C00668,C01172,C00092	HMDB01401	259.021	9.72	1.064	-1.282	-0.208	0.426
375	1*H*-Imidazole-4-propionic acid	No ID	No ID	141.066	9.02	1.048	-1.217	-0.367	0.535
376	Fructose 6-phosphate	C05345,C00085	HMDB00124	259.021	9.78	0.860	-1.242	-0.376	0.758
377	Kynurenine	C00328,C01718	HMDB00684	209.093	11.01	0.892	-0.866	-0.866	0.839
378	Ribose 5-phosphate	C00117	HMDB01548	229.010	10.34	0.978	-0.862	-0.862	0.746
379	Orotic acid	C00295	HMDB00226	155.009	9.77	1.018	-0.859	-0.859	0.699
380	Pipecolic acid	C00408	HMDB00070,HMDB00716,HMDB05960	130.087	11.51	1.128	-0.652	-1.009	0.534
